# Utilizing Telemedicine Applications in Celiac Disease and Other Gluten-Free-Diet-Dependent Conditions: Insights from the COVID-19 Pandemic

**DOI:** 10.3390/healthcare12111132

**Published:** 2024-05-31

**Authors:** Motti Haimi, Aaron Lerner

**Affiliations:** 1 Health Systems Management Department, The Max Stern Yezreel Valley College, Yezreel Valley 1930600, Israel; 2 Rappaport Faculty of Medicine, Technion-Israel Institute of Technology, Haifa 3109601, Israel; 3 Chaim Sheba Medical Center, The Zabludowicz Research Center for Autoimmune Diseases, Ramat Gan 5266202, Israel; aaronlerner1948@gmail.com; 4 Research Department, Ariel University, Ariel 407000, Israel

**Keywords:** celiac disease (CD), gluten, gluten-free diet (GFD), telemedicine, eHealth, COVID-19, GFD-dependent, non-celiac gluten sensitivity (NCGS), compliance, diet-adherence

## Abstract

Background: Globally, approximately 1.4% of people have celiac disease (CD), induced by gluten sensitivity. If left untreated, it causes small intestinal inflammation and villous atrophy, which can result in failure to thrive, anemia, osteoporosis, malabsorption, and even malignancy. The only treatment option available is a gluten-free diet (GFD). Few studies have looked at the role and perception of telehealth in relation to CD and selective nutrition both before and after the COVID-19 pandemic. Aim: Our goal was to screen and investigate the research conducted both before and after the COVID-19 pandemic concerning the utilization of telehealth applications and solutions in CD and other GFD-dependent circumstances. Methods: We employed a narrative review approach to explore articles that were published in scholarly journals or organizations between the years 2000 and 2024. Only English-language publications were included. PubMed and Google Scholar searches were mainly conducted using the following keywords: telemedicine, telehealth, telecare, eHealth, m-health, COVID-19, SARS-CoV-2, celiac disease, and gluten-free diet (GFD). Manual searches of the references in the acquired literature were also carried out, along with the authors’ own personal contributions of their knowledge and proficiency in this field. Results: Only a few studies conducted prior to the COVID-19 outbreak examined the viewpoints and experiences of adult patients with CD with relation to in-person clinic visits, as well as other options such as telehealth. The majority of patients believed that phone consultations were appropriate and beneficial. Video conferencing and telemedicine became more popular during the COVID-19 pandemic, demonstrating the effectiveness of using these technologies for CD on a global basis. In recent years, urine assays for gluten identification have become accessible for use at home. These tests could be helpful for CD monitoring with telemedicine assistance. Conclusions: The extended knowledge gathered from the COVID-19 pandemic is expected to complement pre-COVID-19 data supporting the usefulness of telemedicine even after the emergent pandemic, encouraging its wider adoption in standard clinical practice. The monitoring and follow-up of CD patients and other GFD-dependent conditions can greatly benefit from telemedicine.

## 1. Introduction

Celiac disease (CD), an autoimmune condition, affects approximately 1.4% of people worldwide.

It is identified by gluten sensitivity. The target organ is the small intestine, where inflammation injures the mucosa, resulting in a variety of gastrointestinal (GI) and non-GI symptoms [1,2,3,4]. 

Untreated, it induces inflammation in the small intestine, resulting in villous atrophy, which can lead to chronic diarrhea, failure to thrive, malabsorption, anemia, osteoporosis, nutritional deficiencies, and even cancer [5,6,7]. The sole therapeutic option is to consume a gluten-free diet (GFD) [5,6,7,8]. 

It is generally acknowledged that following a GFD may be difficult; it calls for motivation, knowledge, and altered behavior. The GFD adherence rate might vary from 42% to 91%, depending on the patient category and recruitment strategy. Strict adherence to a gluten-free diet (GFD) is difficult for a variety of reasons, including a lack of awareness, sub-standard labeling, and the high price of gluten-free goods [9,10].

Expert dietitian-led individualized instruction is regarded as the gold standard of care. It takes a lot of money and effort, however. In order to get around these restrictions, other teaching strategies, such as group instruction, have been researched [10,11,12,13]. It should be mentioned that GFD has its side effects [14,15,16] and gluten avoidance can prevent its detrimental effect, not only in celiac patients [16,17]. Improved patient knowledge and comprehensive nutritional education will improve patient compliance [9,15,16,17,18]. 

Across the world, a variety of techniques are employed to help medical professionals monitor and assess patients. These techniques include nutritional and psychological counseling, one-on-one consultations, group meetings, phone clinics, web interfaces, and text messaging, with varying degrees of success documented [19,20].

Educating celiac patients about gluten-free foods, medications, and consumer goods was thought to be a key strategy for improving their adherence to the GFD and reducing the symptoms of the illness [9,14,21]. 

Transmitting health messages via cellphones has increased recently as a result of the rise in smartphone use worldwide. Utilizing mobile technologies to improve health outcomes is a suitable strategy known as mobile health, or mHealth. This approach is accessible, affordable, and simple to implement. In several studies, this strategy has been employed to encourage behaviors relevant to health [22,23,24,25,26,27,28,29,30,31]. 

Dietary therapy has demonstrated the benefits of smartphone applications for weight loss programs [32,33,34,35,36] and nutrition education for many illnesses, including diabetes [37], kidney ailments [38], and cardiovascular disease [39].

Telephone and smartphone applications have also been reported to be effective for celiac patients [40,41]. 

Dowd et al. demonstrated that one-month use of the application had a considerably greater favorable effect on GI symptoms in celiac patients than usual therapy [42]. 

In addition to the gluten content of meals, patients can receive information such as gluten-related labeling and a gluten-free drug list via applications. Previously, a lack of understanding regarding the gluten content of commercial foods and medications was noted as a barrier to GFD compliance [21].

The COVID-19 pandemic has had a considerable detrimental impact on world health and the economy, and it has prompted fresh worries among CD patients [43]. Celiac disease, an immune-mediated ailment, has been related to a higher risk of bacterial pneumonia [44], as well as frequent viral infections including influenza [45] and herpes zoster [46]. These findings have sparked worries about whether celiac patients are more susceptible to contract COVID-19, which can have severe consequences. The pandemic could also potentially harm people who are genetically prone to celiac disease, as viral infections have been found to cause an immune response to dietary antigens [47,48,49].

Moreover, individuals with CD must deal with pressing issues, including the potential loss of access to medical care and gluten-free food alternatives. Telemedicine has quickly emerged as the main instrument for outpatient assessment as a result of this problem [50]. 

Only a small number of studies have examined the function and perception of telehealth, in gastroenterology in general, and in CD and nutrition in particular [51,52,53] before and during the COVID-19 pandemic. These studies typically yielded positive results in favor of telemedicine, with televisits with patients in their homes appearing acceptable for nutritional counseling due to the ease with which patients can submit dietary advice during such in-person consultations [54].

## 2. Methods

### 2.1. Search Terms

The following keywords were used to search PubMed and Google Scholar for this narrative review: telemedicine, telehealth, telecare, eHealth, m-health, celiac disease, COVID-19, SARS-CoV-2, gluten-free diet (GFD).

In addition, manual searches of references in the obtained literature were conducted. We also discussed with professionals and experts in the fields of telemedicine and celiac disease. In addition to producing numerous assessments of the relevant literature, we contributed our own expertise and experience in those domains. Table 1 provides a summary of the search terms.

### 2.2. Selection Criteria

Only English-language publications that were published in scholarly journals or organizations between January 2000 and April 2024 were included.

All types of articles were considered, including original articles, reports of randomized clinical trials, observational studies, and editorials, reviews, or essays by key opinion leaders. 

## 3. Results

### 3.1. Celiac Disease and Other GFD-Compatible Conditions

As previously mentioned, celiac disease (CD) is an immune-mediated illness that affects people who are genetically susceptible (HLA DQ2 and DQ8 genotypes). It is induced by consuming gluten, which is found in grains including wheat, barley, and rye. About 1.4% of people worldwide are affected with CD, and reports of its prevalence in the US and Europe have been rising over time [55,56,57,58]. Furthermore, it seems to be increasing, with a patchy distribution and areas in Saudi Arabia where the sero-prevalence might reach up to 3% [59,60].

The only proven method for treating CD patients’ symptoms, returning their immune systems to normal, as well as the normalization of serological markers and healing their intestinal mucosa, is a life-long gluten-free diet (GFD) [9,14,61,62,63,64].

The histologic and serological criteria of classic CD are not met in some borderline instances that have received a lot of attention lately, but it appears that gluten may contribute to the symptoms. A gluten-free diet appears to help this diverse group, known as “non-celiac gluten sensitivity” (NCGS), at least symptomatically. Their histopathologic analysis also reveals milder villus architectural alterations. These symptoms are typically milder than those of classic CD [65,66,67,68,69]. Survey study data indicate that the prevalence rates of NCGS range from 0.49% to 14.9%, exceeding nearly all estimates of CD or WA (wheat allergy) prevalence rates [70].

A subset of this group, known as lymphocytic doudenosis, is identified by elevated intraepithelial lymphocytes (IELs) in the small intestine biopsy, without any other histopathologic characteristics of the CD and negative serology [71]. 

In addition to gluten proteins, other parts of wheat have been suggested by researchers to trigger symptoms in NCGS patients and activate the innate immune system [72,73].

Furthermore, a few trials have demonstrated relief in individuals with non-specific enteropathies, dyspepsia, or IBS-like symptoms after a GFD, indicating that a GFD may have uses beyond treating people with classic CD [74,75,76]. Notably, GFD was recently reported to benefit patients with non-celiac autoimmune conditions [17].

It is generally acknowledged that the consumption of grains such as wheat, rye, and barley by individuals in whom CD has been conclusively ruled out can be linked to extraintestinal symptoms such as fatigue, as well as typical IBS-like symptoms such as bloating, abdominal pain, and irregular bowel habits. Consequently, diagnoses of NCGS have been performed for individuals who do not have wheat allergy or CD but who experience gastrointestinal symptoms similar to IBS after eating gluten-containing foods and who see improvements in these symptoms after following a GFD [77].

According to estimates, the prevalence of NCGS ranges from 0.6% to 13.0%. Wheat allergy, and gluten ataxia, are other illnesses that have benefited from GFD. However, a lot of people would rather limit or completely avoid consuming gluten. Up to 50% of some groups, including athletes, report varying degrees of adherence to a GFD [78,79]. 

Atopic illnesses and nongrain food allergies in children have been found to be more common in patients with NCGS and IBS symptoms [80].

The outcomes of the first trial using a GFD for fibromyalgia were published in a recent study by Slim and colleagues [81]. In this study, over the course of 24 weeks, 75 adult patients with fibromyalgia were randomized to either a GFD or a hypocaloric diet (HCD). Similar positive effects in lowering the symptoms of gluten sensitivity and other secondary outcomes were linked to both dietary treatments.

The effects of a GFD in patients with endometriosis and persistent pelvic discomfort have been assessed in two studies. Following the application of a GFD for six or twelve months, both trials reported an improvement in pain scores [82,83].

### 3.2. Telehealth Application for Celiac Patients

Very few studies in the literature have assessed the function and perception of telehealth, specifically televisits in gastroenterology and even fewer in celiac disease, both before and after the COVID-19 pandemic.

None of these studies explore the perspective of patients’ trust in telemedicine and televisits, despite the fact that similar studies conducted in other disciplines have generally shown positive results in terms of satisfaction and cost-effectiveness [51,52,53,54].

Across the world, a variety of techniques are employed to allow medical professionals to monitor and assess patients, such as individual consultations, group meetings, phone clinics, and web-based tools [20,21]. 

According to Kurien et al. [84], efforts must be made to develop a financially sensible method of providing a CD follow-up treatment that is well-received by patients and medical professionals. 

Thirty-four persons with CD participated in qualitative interviews [75] to discuss their preferences for the kind of intervention that would be designed to increase dietary adherence. In addition to being easy, flexible, and convenient, the participants thought that telephone clinics were just as helpful as in-person clinics when it came to GF dietary adherence. This study showed that a six-month intervention using a telephone clinic increases GF dietary adherence. The participants’ understanding of nutrition increased, especially with regard to their awareness of what gluten-free items they could eat. The study also showed that individuals who did not strictly follow a GFD needed to be regularly followed up with because the improvements showed maximum benefit after six months [85]. 

Muhammad et al. conducted another study to learn more about the perspectives and experiences of adult CD patients regarding in-person clinic visits and other options such as telehealth. Based on simplicity and convenience, most patients thought that telephone consultations were acceptable and useful [86].

Nikniaz et al. [41] created a Persian-language application for CD patients and evaluated the impact of a three-month educational intervention given via smartphone application on gastrointestinal symptom rating scale (GSRS) score in patients with CD in comparison with routine care. 

The findings showed that, in comparison to standard clinic teaching, utilizing a smartphone application to educate patients with CD significantly improved their symptoms of indigestion. The CD-GSRS mean score (*p* = 0.001) and the indigestion sub score (*p* < 0.001) in the intervention group were both significantly lower than the baseline.

### 3.3. The Effect of the COVID-19 Pandemic

The COVID-19 pandemic has led to an increase in the use of video conferencing and telemedicine in place of in-person consultations, which has proven the efficacy of employing these technologies for CD on a global scale [87].

Telemedicine was adopted for CD patients during the countrywide lockdown during the COVID-19 pandemic in Italy [54]. Phone calls were conducted between March 2020 and May 2020 for follow-up appointments that were previously planned but never completed at the tertiary referral center (Centre for Prevention and Diagnosis of Celiac Disease in Milan, Italy). In addition to phone calls, televisits were suggested for any patient exhibiting symptoms (such as diarrhea, abdominal pain, or weight loss) or for those exhibiting changed blood test results. Patients were also given the option of televisits for nutritional counseling on GFD and CD. Depending on the preferences of each patient, video calling services from Microsoft Teams or Google (Hangouts or Meet) were employed. According to this study, telemedicine was a viable option, and CD patients had a positive attitude towards it. Most patients trusted the combined gastroenterological and nutritional televisits. Gluten detection tests were shown to be useful in helping patients and caregivers verify GFD compliance from a distance.

Researchers have worked to determine whether CD patients are more likely to experience severe COVID-19 outcomes during the pandemic. Undoubtedly, the pandemic has altered how CD is managed; for example, lockdowns have necessitated the use of new technologies to provide medical care during this time [88].

It was first thought that those who have autoimmune diseases including type 1 diabetes and CD would be more susceptible to catching COVID-19. Furthermore, there was a concern that these people would experience more serious COVID-19-infection-related outcomes [89].

A study conducted in Italy in March 2020 found that patients with celiac disease who are older or have other chronic illnesses perceive a larger risk of contracting COVID-19 infection [52]. Nevertheless, elderly adults may benefit from an increased awareness of celiac disease [90].

CD has been associated with an increased risk of respiratory infection outcomes, such as pneumococcal pneumonia and influenza, as well as a greater mortality rate from respiratory causes [44,91,92].

Hospitalization and death rates for people with CD were comparable to those in the general population, according to studies carried out before COVID-19 vaccinations were made available. In addition, CD patients did not have higher COVID-19-related morbidity or mortality, according to early American research [93,94,95].

In conclusion, there is currently universal agreement that individuals with celiac disease do not have an increased chance of developing symptomatic COVID-19, despite initial worries that this patient population might be more affected by the virus than the general population [89].

In relation to obtaining gluten-free food and adhering to a GFD, early in 2020, as COVID-19 spread over the world, many households bought a lot of long-lasting products, which had a big impact on access to particular products. Furthermore, there was restricted entry to restaurants due to strict lockdowns in numerous nations. Due to their dietary limitations, those with CD were particularly burdened by this, and compared to the general population, people with CD reported higher degrees of food insecurity [96]. 

The purposeful consumption of gluten-containing items increased as a result of a decrease in the availability of gluten-free meals, according to a study conducted on American families [97]. More recently diagnosed CD patients had more trouble following a GFD, according to a Turkish study [98].

People in Chile stated that maintaining a GFD was challenging because of rising prices for gluten-free goods, job losses, and food shortages [99].

Two significant ways that environmental factors can impact a GFD were brought to light by the COVID-19 pandemic. Some reported sticking to a GFD more closely since they were unable to eat during the severe lockdown [100]. On the other hand, other CD patients reported more difficulty paying for gluten-free food and more obstacles to sticking to a GFD [99].

In a study conducted by Siniscalchi et al. [52], the researchers sought to understand how persons with CD from Veneto and Campania, Italy, for whom food supplies are a daily concern, saw the COVID-19 pandemic during the lockdown. Significant percentages of respondents to a quality-of-life questionnaire related to CD stated that they felt generally intimidated and depressed as a result of their condition. Concerns about their celiac illness, which increases the risk of infection and serious CD consequences, were more prevalent among older responders. However, the main finding was that individuals with CD did not believe they were at a heightened risk of contracting the SARS-CoV-2 virus. Another noteworthy conclusion is that the majority of CD patients were content with remote consultations and were not concerned about the lack of gluten-free food in stores or pharmacies. According to the findings, CD healthcare may eventually adopt remote consulting as normal practice.

During the first phase of the COVID-19 pandemic, a cross-sectional questionnaire-based study involving 37 Dutch pediatric patients with CD and their parents was conducted to learn more about the experiences of children with CD and their families regarding their GFD, symptoms, and CD management. The majority said that eating more meals at home made it simpler to stick to the diet during the pandemic and reported strong compliance with the GFD. Some found that online purchasing offered a wider selection of GF items, which could increase the GFD’s cost. In terms of overall eating patterns, 21.6% reported a healthier pattern, compared to 37.8% and 10.8% who, respectively, had fewer fruits and vegetables and more unhealthy snacks than usual over the pandemic [101].

In certain cases, the pandemic also affected the diagnosis of CD. The British Society for Gastroenterology released a non-biopsy-based protocol for CD diagnosis in 2020 in an attempt to reduce exposure to COVID-19. Villus atrophy on endoscopy appears to be substantially correlated with laboratory data, particularly increased IgA tissue transglutaminase levels 10 times the upper limit of normal or above. Those guidelines state that a positive endomysial antibody and a highly elevated IgA tissue transglutaminase (tTG; ≥10 times the upper limit of normal) in individuals under 55 years of age can be regarded as a confirmed diagnosis of CD [102].

### 3.4. Future Use in a Non-Pandemic Scenario

Even after the emergency pandemic, the experience gained from the COVID-19 pandemic is likely to support pre-COVID-19 evidence regarding the efficacy and good performance of telemedicine and encourage its wider adoption in routine clinical practice.

During the pandemic, telehealth made it possible for those with celiac disease to obtain dietary counseling, which is essential in adopting GFD. In places with limited healthcare resources, telehealth will probably make dietitians more accessible. During the pandemic, telehealth had additionally made high-quality gastrointestinal treatment more accessible. Given its widespread use, telemedicine is expected to become more prevalent in medicine as long as technological advancements keep pace. Using telemedicine, a larger patient group may be able to receive specialist care [103].

Early in the COVID-19 pandemic, a lack of access to endoscopy resulted in modifications to the diagnostic standards for celiac disease, which are probably going to be followed in non-pandemic times as well. Outside of this context, a non-biopsy method to the diagnosis of adult CD is not commonly used; however, this may be beginning to change. A very elevated tTG IgA in adults (10 times the upper normal limit) had a 95% positive predictive value, according to a multicenter investigation [104,105].

Technology for self-monitoring health may have become more popular as a result of the COVID-19 pandemic. Urine assays that can be performed at home to identify gluten have been available in recent years. The presence of gluten immunogenic peptides (GIPs) in the urine and stool may make it possible to monitor compliance and identify individuals who were not following a GFD. These tests might be useful for telemedicine-assisted CD monitoring [54,106,107].

Individuals who have CD must deal with pressing issues such as the potential loss of access to healthcare and gluten-free dietary alternatives. Due to this latter problem, telemedicine has quickly emerged as the main instrument for outpatient assessment. For this patient group, telemedicine is a popular technology that provides a means of giving people who live outside of cities access to subspecialists in CD. Telemedicine holds great potential in the monitoring and follow-up of these patients, especially considering the relative lack of specialist knowledge in the field in general, and qualified dietitians in particular [50].

## 4. Discussion

Genetically vulnerable people worldwide are affected with celiac disease (CD), a life-long, gluten-sensitive autoimmune illness of the small intestine. In people with CD, symptoms may be extraintestinal, gastrointestinal, or absent. Diarrhea, steatorrhea, and weight loss from malabsorption are among the gastrointestinal symptoms that are considered classical. Anemia, osteoporosis, dermatitis herpetiformis, neurological issues, and hypoplasia of the tooth enamel are extraintestinal or atypical symptoms that over 50% of CD patients experience. Genetic and immunological bases contribute to the diverse clinical picture of CD; factors influencing the disease’s clinical expression include the age at onset, degree of mucosal injury, dietary habits, and gender [108,109].

While the amount of people worldwide who suffer from celiac disease is only 1.4%, there are locations where this percentage is much higher. The area of birth, a family history of celiac disease, female sex, and the amount of gluten taken in early life are significant risk factors for the development of this autoimmune illness. Roughly twice as many women as men are impacted. The increased incidence of celiac disease and the number of cases of gluten sensitivity in the general population are worrying [2,3,4,108,110].

A lifetime GFD is the only established way to alleviate the symptoms of CD patients, restore their immune systems to normal, and heal their intestinal mucosa [61,62]. 

Telemedicine is widely acknowledged as a useful tool with enormous potential. Individual consultations, group meetings, phone clinics, and web-based tools are among the strategies used globally by medical professionals to monitor and assess patients [19,20]. 

However, its use in daily clinical practice may still be limited because most patient communication takes place via emails and texts, which patients frequently find unsatisfactory because of the offline interaction and response latency [111,112]. 

Remote monitoring may be crucial given that the only treatment available to individuals with CD (except from those with refractory celiac disease) is rigorous adherence to a GFD. However, it has taken a global epidemic to spur the widespread adoption of telemedicine, even though it has long shown promise in the treatment of CD [54].

Health care professionals can now communicate with patients over long distances using synchronous and asynchronous modalities, primarily live videoconferencing that enables interactive consultation and prompt interventions, thanks to sophisticated communication technologies, most commonly computers and mobile phones. However, patients who are unfamiliar with digital technologies may hinder the spread of telemedicine [113].

Furthermore, the necessity for improved diagnostic standards, the scarcity of specialists, and the high cost of gluten-free food make the management of this patient group problematic. In many European nations, the condition progresses for an average of eight years before a diagnosis is made, during which time the patient’s body deteriorates due to a variety of factors and cannot be healed. Thus, suggestions to use cellphones to monitor the amount of gluten in food products and to contact specialists give many patients—especially those who live in small towns far from specialist centers and those with low socioeconomic status—great hope for a better quality of life [21,50,114,115,116].

Research has shown that CD patients have a favorable attitude toward telemedicine and that it was a feasible option. A vital requirement for feeling confident in suggesting this strategy and seeing it through to effective implementation is the good trust rate in televisits among CD patients [51,52,53,54,85,86]. 

In-person visits were kept for people who were either incapable of using technology or did not have access to the internet or other electronic devices. As an alternative, the patient’s caregiver could provide support for the subset of patients who are ultimately ineligible for remote help [113].

Nevertheless, the experience gained from the COVID-19 pandemic is likely to support pre-COVID-19 evidence regarding the efficacy and good performance of telemedicine and encourage its wider integration into routine clinical practice in general, and in CD patients in particular [52,54,87,88].

Furthermore, in certain situations, self-administered and point-of-care assays (such urinary GIP, or gluten immunogenic peptides) that monitor GFD may be a valuable resource for patients with CD.

They have been shown to be an invaluable tool for celiac patients to monitor their GFD, enhancing adherence and ensuring that no unintentional gluten ingestion occurs. Even during distant televisits, these point-of-care tests can be used as simple, affordable, accurate, and reliable instruments to confirm potential gluten contamination [106,117,118]. 

Since a GFD appears to help also in group of conditions, known as “non-celiac gluten sensitivity” (NCGS), these people can also benefit from the telehealth solutions described here [65,66,67,68,69,70,71,72,73,74,75,76,77].

Regardless of the patients’ financial situation, the telehealth systems discussed here offer a great basis for creating and enduringly implementing an efficient system into the care of patients with gluten sensitivity and celiac disease around the globe. Furthermore, the task of specialists in the treatment of this patient group will be made easier by such a system [40,86,114,119]. 

## 5. Conclusions

Across the world, a variety of techniques are employed to allow medical professionals to monitor and assess patients with CD. 

During the COVID-19 pandemic, people with CD experienced urgent concerns to address, such as the loss of access to healthcare and gluten-free food options. Telemedicine has therefore swiftly become the primary tool for outpatient assessment. 

The extended knowledge gathered from the COVID-19 pandemic is expected to complement pre-COVID-19 data supporting the usefulness of telemedicine even after the emergent pandemic, encouraging its wider adoption in standard clinical practice. 

The monitoring and follow-up of these patients can greatly benefit from telemedicine, especially in light of the relative dearth of specialized knowledge in this area.

## 6. Limitations

Although this narrative review was thorough, as with all narrative evaluations, selection bias cannot be completely eliminated.

Since we mainly included publications that discussed favorable telemedicine experiences in CD, this could potentially have led to confirmation bias.

## Figures and Tables

**Table 1:** Search criteria and sources used for the study.

Type of Review	Narrative Review
**Period**	2000 to 2024
**Keywords used**	Telemedicine, telehealth, telecare, eHealth, m-health, celiac disease, COVID-19, SARS-CoV-2, gluten-free diet (GFD).
**Sources**	PubMed, Google Scholar. Manual searches of the obtained literature’s references. Discussion with professionals and experts in the fields of telemedicine and celiac disease. Bringing our individual experiences and knowledge of that fields’ topics.
**Language**	English only
**Types of articles**	Original articles; Reports of randomized clinical trials; Observational studies; Editorials, reviews, and essays by key opinion leaders.

## Data Availability

All data are available on request.

## References

[1] Lerner A., Matthias T., Wusterhausen P. (2019). Autoimmunity in celiac disease: Extra-intestinal manifestations. Autoimmun. Rev..

[2] Makovicky P., Makovicky P., Caja F., Rimarova K., Samasca G., Vannucci L. (2020). Celiac disease and gluten-free diet: Past, present, and future. Gastroenterol. Hepatol. Bed Bench.

[3] Singh P., Arora A., Strand T.A., Leffler D.A., Catassi C., Green P.H., Kelly C.P., Ahuja V., Makharia G.K. (2018). Global Prevalence of Celiac Disease: Systematic Review and Meta-analysis. Clin. Gastroenterol. Hepatol..

[4] Reilly N.R., Fasano A., Green P.H.R. (2012). Presentation of celiac disease. Gastrointest. Endosc. Clin. N. Am..

[5] Mahadev S., Laszkowska M., Sundström J., Björkholm M., Lebwohl B., Green P.H.R., Ludvigsson J.F. (2018). Prevalence of celiac disease in patients with iron deficiency Anemia—A systematic review with meta-analysis. Gastroenterology.

[6] Meyer D., Stavropolous S., Diamond B., Shane E., Green P.H. (2001). Osteoporosis in a North American adult population with celiac disease. Am. J. Gastroenterol..

[7] Emilsson L., Semrad C., Lebwohl B., Green P.H.R., Ludvigsson J.F. (2020). Risk of small bowel adenocarcinoma, adenomas, and carcinoids in a nationwide cohort of individuals with celiac disease. Gastroenterology.

[8] Rubio-Tapia A., Hill I.D., Kelly C.P., Calderwood A.H., Murray J.A. (2013). ACG clinical guidelines: Diagnosis and management of celiac disease. Am. J. Gastroenterol..

[9] Samasca G., Lerner A., Girbovan A., Sur G., Lupan I., Makovicky P., Matthias T., Freeman H.J. (2017). Challenges in gluten-free diet in coeliac disease: Prague consensus. Eur. J. Clin. Investig..

[10] Muhammad H., Reeves S., Jeanes Y.M. (2019). Identifying and improving adherence to the gluten-free diet in people with coeliac disease. Proc. Nutr. Soc..

[11] Hall N.J., Rubin G., Charnock A. (2009). Systematic review: Adherence to a gluten-free diet in adult patients with coeliac disease. Aliment. Pharmacol. Ther..

[12] Akbari Namvar Z., Mahdavi R., Shirmohammadi M., Nikniaz Z. (2021). The effect of group-based education on knowledge and adherence to a gluten-free diet in patients with celiac disease: Randomized controlled clinical trial. Int. J. Behav. Med..

[13] Sainsbury K., Mullan B., Sharpe L. (2013). A randomized controlled trial of an online intervention to improve gluten-free diet adherence in celiac disease. Off. J. Am. Coll. Gastroenterol. ACG.

[14] Lerner A., Matthias T. (2017). Gluten-free Diet—Tough Alley in Torrid Time. Internat J. Celiac Dis..

[15] Lerner A., O’Bryan T., Matthias T. (2019). Navigating the Gluten-Free Boom: The Dark Side of Gluten Free Diet. Front. Pediatr..

[16] Lerner A., Shoenfeld Y., Matthias T. (2017). Adverse effects of gluten ingestion and advantages of gluten withdrawal in nonceliac autoimmune disease. Nutr. Rev..

[17] Lerner A., Freire de Carvalho J., Kotrova A., Shoenfeld Y. (2022). Gluten-free diet can ameliorate the symptoms of non-celiac autoimmune diseases. Nutr. Rev..

[18] Bascunan K.A., Vespa M.C., Araya M. (2017). Celiac disease: Understanding the gluten-free diet. Eur. J. Nutr..

[19] Sainsbury K., Mullan B., Sharpe L. (2015). Predicting intention and behaviour following participation in a theory-based intervention to improve gluten free diet adherence in coeliac disease. Psychol. Health.

[20] Kallos S., Jeanes Y. (2020). Cross-Sectional survey of the dietetic provision for adults with coeliac disease in the UK. J. Hum. Nutr. Diet..

[21] Rajpoot P., Sharma A., Harikrishnan S., Baruah B.J., Ahuja V., Makharia G.K. (2015). Adherence to gluten-free diet and barriers to adherence in patients with celiac disease. Indian. J. Gastroenterol..

[22] LeBeau K., Huey L.G., Hart M. (2019). Assessing the quality of mobile apps used by occupational therapists: Evaluation using the user version of the mobile application rating scale. JMIR mHealth uHealth.

[23] Kumar S., Nilsen W.J., Abernethy A., Atienza A., Patrick K., Pavel M., Riley W.T., Shar A., Spring B., Spruijt-Metz D. (2013). Mobile health technology evaluation: The mHealth evidence workshop. Am. J. Prev. Med..

[24] Díaz-Hellín P., Fontecha J., Hervás R., Bravo J. (2015). NFC as a childhood obesity treatment tool. J. Med. Syst..

[25] El-Gayar O., Timsina P., Nawar N., Eid W. (2013). Mobile applications for diabetes self-management: Status and potential. J. Diabetes Sci. Technol..

[26] Semper H., Povey R., Clark-Carter D. (2016). A systematic review of the effectiveness of smartphone applications that encourage dietary self-regulatory strategies for weight loss in overweight and obese adults. Obes. Rev..

[27] Cook K.A., Modena B.D., Simon R.A. (2016). Improvement in asthma control using a minimally burdensome and proactive smartphone application. J. Allergy Clin. Immunol. Pract..

[28] Licskai C.J., Sands T.W., Ferrone M. (2013). Development and pilot testing of a mobile health solution for asthma self-management: Asthma action plan smartphone application pilot study. Can. Respir. J..

[29] Zeltzer D., Einav L., Rashba J., Waisman Y., Haimi M., Balicer R.D. (2023). Adoption and utilization of device-assisted telemedicine. J. Health Econ..

[30] Haimi M., Goren U., Grossman Z. (2024). Barriers and challenges to telemedicine usage among the elderly population in Israel in light of the COVID-19 era: A qualitative study. Digit. Health.

[31] Haimi M., Sergienko R. (2024). Adoption and Use of Telemedicine and Digital Health Services Among Older Adults in Light of the COVID-19 Pandemic: Repeated Cross-Sectional Analysis. JMIR Aging.

[32] Carter M.C., Burley V.J., Nykjaer C., Cade J.E. (2013). Adherence to a smartphone application for weight loss compared to website and paper diary: Pilot randomized controlled trial. J. Med. Internet Res..

[33] Gilliland J., Sadler R., Clark A., O’Connor C., Milczarek M., Doherty S. (2015). Using a smartphone application to promote healthy dietary behaviours and local food consumption. BioMed Res. Int..

[34] Laing B.Y., Mangione C.M., Tseng C.H., Leng M., Vaisberg E., Mahida M., Bholat M., Glazier E., Morisky D.E., Bell D.S. (2014). Effectiveness of a smartphone application for weight loss compared with usual care in overweight primary care patients: A randomized, controlled trial. Ann. Intern. Med..

[35] Stephens J.D., Yager A.M., Allen J. (2017). Smartphone technology and text messaging for weight loss in young adults: A randomized controlled trial. J. Cardiovasc. Nurs..

[36] Allen J.K., Stephens J., Dennison Himmelfarb C.R., Stewart K.J., Hauck S. (2013). Randomized controlled pilot study testing use of smartphone technology for obesity treatment. J. Obes..

[37] Holmen H., Torbjørnsen A., Wahl A.K., Jenum A.K., Småstuen M.C., Arsand E., Ribu L. (2014). A mobile health intervention for self-management and lifestyle change for persons with type 2 diabetes, part 2: One-year results from the Norwegian randomized controlled trial RENEWING HEALTH. JMIR mHealth uHealth.

[38] Chang A.R., Bailey-Davis L., Hetherington V., Ziegler A., Yule C., Kwiecen S., Graboski E., Melough M.M., Collins C., Anderson C. (2020). Remote dietary counseling using smartphone applications in patients with stages 1-3a chronic kidney disease: A mixed methods feasibility study. J. Ren. Nutr..

[39] Eyles H., McLean R., Neal B., Jiang Y., Doughty R.N., McLean R., Ni Mhurchu C. (2017). A salt-reduction smartphone app supports lower-salt food purchases for people with cardiovascular disease: Findings from the SaltSwitch randomised controlled trial. Eur. J. Prev. Cardiol..

[40] Muhammad H., Reeves S., Ishaq S., Mayberry J.F., Jeanes Y.M. (2021). Telephone clinic improves gluten free dietary adherence in adults with coeliac disease: Sustained at 6 months. Frontline Gastroenterol..

[41] Nikniaz Z., Namvar Z.A., Shirmohammadi M., Maserat E. (2022). Smartphone Application for Celiac Patients: Assessing Its Effect on Gastrointestinal Symptoms in a Randomized Controlled Clinical Trial. Int. J. Telemed. Appl..

[42] Dowd A.J., Warbeck C.B., Tang K.T., Fung T., Culos-Reed S.N. (2020). MyHealthyGut: Findings from a pilot randomized controlled trial on adherence to a gluten-free diet and quality of life among adults with celiac disease or gluten intolerance. Digit. Health.

[43] Lebwohl B., Sanders D., Green P.H.R. (2018). Celiac disease. Lancet.

[44] Simons M., Scott-Sheldon L.A.J., Risech-Neyman Y. (2018). Celiac disease and increased risk of pneumococcal infection: A systematic review and meta-analysis. Am. J. Med..

[45] Kårhus L.L., Gunnes N., Størdal K. (2018). Influenza and risk of later celiac disease: A cohort study of 2.6 million people. Scand. J. Gastroenterol..

[46] Ludvigsson J.F., Choung R.S., Marietta E.V. (2018). Increased risk of herpes zoster in patients with coeliac disease—Nationwide cohort study. Scand. J. Public Health.

[47] Bouziat R., Biering S.B., Kouame E. (2018). Murine norovirus infection induces TH1 inflammatory responses to dietary antigens. Cell Host Microbe.

[48] Samasca G., Lerner A. (2021). Celiac disease in COVID-19 pandemic. J. Transl. Autoimmun..

[49] Lerner A. (2020). Are My Patients with Celiac Disease at Higher Risk of COVID-19 Virus?. Int. J. Celiac Dis..

[50] Ines Pinto-Sanchez M., Lebwohl B. (2020). Delivery of care remotely through telemedicine in celiac disease: Thinking beyond COVID-19: Commentary on: “COVID-19 pandemic perception in adults with celiac disease: An impulse to implement the use of telemedicine” by Siniscalchi M et al. Dig Liv Dis 2020;52:1071-5. Dig. Liver Dis..

[51] Vriezinga S., Borghorst A., van den Akker-van Marle E., Benninga M., George E., Hendriks D., Hopman E., de Meij T., van der Meulen-de Jong A., Putter H. (2018). E-Healthcare for celiac disease a multicenter randomized controlled trial. J. Pediatr..

[52] Siniscalchi M., Zingone F., Savarino E.V., D’Odorico A., Ciacci C. (2020). COVID-19 pandemic perception in adults with celiac disease: An impulse to implement the use of telemedicine. Dig. Liver Dis..

[53] Rollo M.E., Hutchesson M.J., Burrows T.L., Krukowski R.A., Harvey J.R., Hoggle L.B., Collins C.E. (2015). Video consultations and virtual nutrition care for weight management. J. Acad. Nutr. Diet..

[54] Costantino A., Roncoroni L., Noviello D., Nandi N., Lombardo V., Scricciolo A., Scaramella L., Vecchi M., Elli L. (2021). Nutritional and Gastroenterological Monitoring of Patients With Celiac Disease During COVID-19 Pandemic: The Emerging Role of Telemedicine and Point-of-Care Gluten Detection Tests. Front. Nutr..

[55] Di Sabatino A., Corazza G.R. (2009). Coeliac disease. Lancet.

[56] Catassi C. (2005). The world map of celiac disease. Acta Gastroenterol. Latinoam..

[57] Rubio-Tapia A., Kyle R.A., Kaplan E.L., Johnson D.R., Page W., Erdtmann F., Brantner T.L., Kim W.R., Phelps T.K., Lahr B.D. (2009). Increased prevalence and mortality in undiagnosed celiac disease. Gastroenterology.

[58] Lohi S., Mustalahti K., Kaukinen K., Laurila K., Collin P., Rissanen H., Lohi O., Bravi E., Gasparin M., Reunanen A. (2007). Increasing prevalence of coeliac disease over time. Aliment. Pharmacol. Ther..

[59] King J.A., Jeong J., Underwood F.E., Quan J., Panaccione N., Windsor J.W., Coward S., DeBruyn J., Ronksley P.E., Shaheen A.A. (2020). Incidence of Celiac Disease Is Increasing Over Time: A Systematic Review and Meta-analysis. Am. J. Gastroenterol..

[60] Ashtari S., Najafimehr H., Pourhoseingholi M.A., Rostami K., Asadzadeh-Aghdaei H., Rostami-Nejad M., Tavirani M.R., Olfatifar M., Makharia G.K., Zali M.R. (2021). Prevalence of celiac disease in low and high-risk population in Asia-Pacific region: A systematic review and meta-analysis. Sci. Rep..

[61] Green P.H., Cellier C. (2007). Celiac disease. N. Engl. J. Med..

[62] Rubio-Tapia A., Murray J.A. (2010). Classification and management of refractory coeliac disease. Gut.

[63] Mazzola A.M., Zammarchi I., Valerii M.C., Spisni E., Saracino I.M., Lanzarotto F., Ricci C. (2024). Gluten-Free Diet and Other Celiac Disease Therapies: Current Understanding and Emerging Strategies. Nutrients.

[64] Niewinski M.M. (2008). Advances in Celiac Disease and Gluten-Free Diet. J. Am. Diet. Assoc..

[65] Hurley J.J., Lee B., Turner J.K., Beale A., Jenkins H.R., Swift G.L. (2012). Incidence and presentation of reported coeliac disease in Cardiff and the Vale of Glamorgan: The next 10 years. Eur. J. Gastroenterol. Hepatol..

[66] Volta U., Villanacci V. (2011). Celiac disease: Diagnostic criteria in progress. Cell Mol. Immunol..

[67] Aziz I., Sanders D.S. (2012). Emerging concepts: From coeliac disease to non-coeliac gluten sensitivity. Proc. Nutr. Soc..

[68] Troncone R., Jabri B. (2011). Coeliac disease and gluten sensitivity. J. Intern. Med..

[69] Mansueto P., Seidita A., D’Alcamo A., Carroccio A. (2014). Non-celiac gluten sensitivity: Literature review. J. Am. Coll. Nutr..

[70] Cárdenas-Torres F.I., Cabrera-Chávez F., Figueroa-Salcido O.G., Ontiveros N. (2021). Non-Celiac Gluten Sensitivity: An Update. Medicina.

[71] Walker M.M., Murray J.A., Ronkainen J., Aro P., Storskrubb T., D’Amato M., Lahr B., Talley N.J., Agreus L. (2010). Detection of celiac disease and lymphocytic enteropathy by parallel serology and histopathology in a population-based study. Gastroenterology.

[72] Talaie R. (2015). Does gluten free diet have more implications than treatment of celiac disease?. Gastroenterol. Hepatol. Bed Bench..

[73] Fasano A., Sapone A., Zevallos V., Schuppan D. (2015). Nonceliac gluten sensitivity. Gastroenterology.

[74] Newnham E.D. (2011). Does gluten cause gastrointestinal symptoms in subjects without coeliac disease?. J. Gastroenterol. Hepatol..

[75] Esteve M., Carrasco A., Fernandēz-Bañares F. (2012). Is a gluten-free diet necessary in Marsh I intestinal lesions in patients with HLADQ2, DQ8 genotype and without gastrointestinal symptoms?. Curr. Opin. Clin. Nutr. Metab. Care..

[76] Rostami Nejad M., Dabiri R., Ehsani-Ardakani M.J., Nazemalhosseini Mojarad E., Derakhshan F., Telkabadi M., Rostami K. (2012). Gluten associated dyspepsia; serology and histological characteristics. Gastroenterol. Hepatol. Bed Bench..

[77] Niland B., Cash B.D. (2018). Health Benefits and Adverse Effects of a Gluten-Free Diet in Non-Celiac Disease Patients. Gastroenterol. Hepatol..

[78] Lis D.M., Stellingwerff T., Shing C.M., Ahuja K.D., Fell J.W. (2015). Exploring the popularity, experiences, and beliefs surrounding gluten-free diets in nonceliac athletes. Int. J. Sport. Nutr. Exerc. Metab..

[79] Aziz I., Dwivedi K., Sanders D.S. (2016). From coeliac disease to noncoeliac gluten sensitivity; should everyone be gluten free?. Curr. Opin. Gastroenterol..

[80] Bucci C., Zingone F., Russo I., Morra I., Tortora R., Pogna N., Scalia G., Iovino P., Ciacci C. (2013). Gliadin does not induce mucosal inflammation or basophil activation in patients with nonceliac gluten sensitivity. Clin. Gastroenterol. Hepatol..

[81] Slim M., Calandre E.P., Garcia-Leiva J.M., Rico-Villademoros F., Molina-Barea R., Rodriguez-Lopez C.M., Morillas-Arques P. (2017). The effects of a gluten-free diet versus a hypocaloric diet among patients with fibromyalgia experiencing gluten sensitivity-like symptoms: A pilot, open-label randomized clinical trial. J. Clin. Gastroenterol..

[82] Marziali M., Venza M., Lazzaro S., Lazzaro A., Micossi C., Stolfi V.M. (2012). Gluten-free diet: A new strategy for management of painful endometriosis related symptoms?. Minerva Chir..

[83] Marziali M., Capozzolo T. (2015). Role of gluten-free diet in the management of chronic pelvic pain of deep infiltrating endometriosis. J. Minim. Invasive Gynecol..

[84] Kurien M., Trott N., Sanders D.S. (2016). Long-Term care for patients with coeliac disease in the UK: A review of the literature and future directions. J. Hum. Nutr. Diet..

[85] Muhammad H., Reeves S., Jeanes Y. (2019). Qualitative Interviews to Explore patient Preference for Health Care Led Interventions to Promote Gluten Free Dietary Adherence. Coeliac, U.K. https://pure.roehampton.ac.uk/portal/en/publications/qualitative-interviews-to-explore-patient-preference-for-health-c.

[86] Muhammad H., Reeves S., Ishaq S., Jeanes Y. (2021). Experiences of Outpatient Clinics and Opinions of Telehealth by Caucasian and South Asian Patients with Celiac Disease. J. Patient Exp..

[87] Alrubaiy L. (2020). Gastroenterology services in the time of COVID-19 pandemic. Frontline Gastroenterol..

[88] Cohen B.S., Lebwohl B. (2023). COVID-19 and celiac disease: A review. Ther. Adv. Gastroenterol..

[89] Zhen J., Stefanolo J.P., Temprano M.P., Seiler C.L., Caminero A., de-Madaria E., Huguet M.M., Santiago V., Niveloni S.I., Smecuol E.G. (2021). Risk perception and knowledge of COVID-19 in patients with celiac disease. World J. Gastroenterol..

[90] Lerner A., Matthias T. (2015). Increased knowledge and awareness of celiac disease will benefit the elderly. Intern. J. Celiac Dis..

[91] Mårild K., Fredlund H., Ludvigsson J.F. (2010). Increased risk of hospital admission for influenza in patients with celiac disease: A nationwide cohort study in Sweden. Am. J. Gastroenterol..

[92] Lebwohl B., Green P.H.R., Söderling J., Roelstraete B., Ludvigsson J.F. (2020). Association between celiac disease and mortality risk in a Swedish population. JAMA.

[93] Mansoor E., Alikhan M.M., Perez J.A., Schlick K., Abou Saleh M., Rubio-Tapia A. (2022). Clinical characteristics, hospitalisation and mortality rates of COVID-19 among patients with coeliac disease in the USA: A multicentre network study. Gut.

[94] Lebwohl B., Larsson E., Söderling J., Roelstraete B., Murray J.A., Green P.H.R., Ludvigsson J.F. (2021). Risk of severe COVID-19 in patients with celiac disease: A population-based cohort study. Clin. Epidemiol..

[95] Hadi Y.B., Sohail A.H., Lakhani D.A., Naqvi S.F., Kupec J.T., Pervez A. (2022). Outcomes of SARS-CoV-2 infection in patients with celiac disease: A multicenter research network study. Ann. Gastroenterol..

[96] Guillaume J.D., Jagai J.S., Makelarski J.A., Abramsohn E.M., Lindau S.T., Verma R., Ciaccio C.E. (2021). COVID-19-related food insecurity among households with dietary restrictions: A national survey. J. Allergy Clin. Immunol. Pract..

[97] Du N., Mehrotra I., Weisbrod V., Regis S., Silvester J.A. (2022). Survey-based study on food insecurity during COVID-19 for households with children on a prescribed gluten-free diet. Am. J. Gastroenterol..

[98] Gokden Y., Hot S., Adas M., Ogutmen Koc D., Atak S., Hot A.B. (2020). Celiac disease and COVID-19 pandemic: Should we worry?. Acta Gastroenterol. Belg..

[99] Bascuñán K.A., Rodríguez J.M., Osben C., Fernández A., Sepúlveda C., Araya M. (2021). Pandemic effects and gluten-free diet: An adherence and mental health problem. Nutrients.

[100] Falcomer A.L., Farage P., Pratesi C.B., Pratesi R., Gandolfi L., Nakano E.Y., Raposo A., Zandonadi R.P. (2021). Health-related quality of life and experiences of brazilian celiac individuals over the course of the SARS-CoV-2 pandemic. Nutrients.

[101] Kreutz J.M., Heynen L., Arayess L., Vreugdenhil A.C.E. (2023). Celiac Disease and the Gluten Free Diet during the COVID-19 Pandemic: Experiences of Children and Parents. Medicina.

[102] British Society of Gastroenterology BSG Interim Guidance 2022: COVID-19 Specific Non-Biopsy Protocol for Those with Suspected Coeliac Disease. https://www.bsg.org.uk/covid-19-advice/covid-19-specific-non-biopsy-protocol-guidance-for-those-with-suspected-coeliac-disease/.

[103] Elli L., Barisani D., Vaira V., Bardella M.T., Topa M., Vecchi M., Doneda L., Scricciolo A., Lombardo V., Roncoroni L. (2020). How to manage celiac disease and gluten-free diet during the COVID-19 era: Proposals from a tertiary referral center in a high-incidence scenario. BMC Gastroenterol..

[104] Penny H.A., Raju S.A., Lau M.S., Marks L.J.S., Baggus E.M.R., Bai J.C., Bassotti G., Bontkes H.J., Carroccio A., Danciu M. (2021). Accuracy of a no-biopsy approach for the diagnosis of coeliac disease across different adult cohorts. Gut.

[105] Rubio-Tapia A., Hill I.D., Semrad C., Kelly C.P., Greer K.B., Limketkai B.N., Lebwohl B. (2023). American college of gastroenterology guidelines update: Diagnosis and management of celiac disease. Am. J. Gastroenterol..

[106] Moreno M.L., Cebolla Á., Muñoz-Suano A., Carrillo-Carrion C., Comino I., Pizarro Á., León F., Rodríguez-Herrera A., Sousa C. (2017). Detection of gluten immunogenic peptides in the urine of patients with coeliac disease reveals transgressions in the gluten-free diet and incomplete mucosal healing. Gut.

[107] Ciacci C., Gagliardi M., Siniscalchi M., Ruotolo M., Santonicola A., Hajji N., Zingone F. (2021). Gluten immunogenic peptides (GIP) point-of-care urine test in coeliac disease follow-up before and during the COVID-19 lockdown in Italy. Clin. Exp. Gastroenterol..

[108] Gujral N., Freeman H.J., Thomson A.B. (2012). Celiac disease: Prevalence, diagnosis, pathogenesis and treatment. World J. Gastroenterol..

[109] Bai D., Brar P., Holleran S., Ramakrishnan R., Green P.H. (2005). Effect of gender on the manifestations of celiac disease: Evidence for greater malabsorption in men. Scand. J. Gastroenterol..

[110] Catassi C., Yachha S.K., Fasano A., Troncone R., Branski D. (2008). The global village of celiac disease. Frontiers in Celiac Disease.

[111] Daschle T., Dorsey E.R. (2015). The return of the house call. Ann. Intern. Med..

[112] Welch B.M., Harvey J., O’Connell N.S., McElligott J.T. (2017). Patient preferences for direct-to-consumer telemedicine services: A nationwide survey. BMC Health Serv. Res..

[113] Wilson L.S., Maeder A.J. (2015). Recent directions in telemedicine: Review of trends in research and practice. Healthc. Inform. Res..

[114] Hanci O., Jeanes Y.M. (2019). Are gluten-free food staples accessible to all patients with coeliac disease?. Frontline Gastroenterol..

[115] Alavinejad P., Hajiani E., Masjedizadeh R., Hashemi S.J., Faramarzi M., Sebghatollahi V., Shayesteh A.A., Kadkhodae A., Jasemi Zergani F., Asghari S. (2014). Epidemiologic and demographic survey of celiac disease in khuzestan province. Middle East. J. Dig. Dis..

[116] Mustalahti K., Catassi C., Reunanen A., Fabiani E., Heier M., McMillan S., Murray L., Metzger M.-H., Gasparin M., Bravi E. (2010). Prevalence of celiac disease in Europe: Results of a centralized, international mass screening project. Ann. Med..

[117] Elli L., Bascuñán K., di Lernia L., Bardella M.T., Doneda L., Soldati L., Orlando S., Ferretti F., Lombardo V., Barigelletti G. (2020). Safety of occasional ingestion of gluten in patients with celiac disease: A real-life study. BMC Med..

[118] Soler M., Estevez M.C., Moreno M.L., Cebolla A., Lechuga L.M. (2016). Label-free SPR detection of gluten peptides in urine for non-invasive celiac disease follow-up. Biosens. Bioelectron..

[119] Wieser H., Ruiz-Carnicer Á., Segura V., Comino I., Sousa C. (2021). Challenges of Monitoring the Gluten-Free Diet Adherence in the Management and Follow-Up of Patients with Celiac Disease. Nutrients.

